# Adenoviral Vectors Armed with Cell Fusion-Inducing Proteins as Anti-Cancer Agents

**DOI:** 10.3390/v9010013

**Published:** 2017-01-19

**Authors:** Joshua Del Papa, Robin J. Parks

**Affiliations:** 1 Regenerative Medicine Program, Ottawa Hospital Research Institute, Ottawa, ON K1H 8L6, Canada; jdelpapa@ohri.ca; 2 Department of Biochemistry, Microbiology, and Immunology, Faculty of Medicine, University of Ottawa, Ottawa, ON K1N 6N5, Canada; 3 Centre for Neuromuscular Disease, University of Ottawa, Ottawa, ON K1N 6N5, Canada; 4 Department of Medicine, University of Ottawa, Ottawa, ON K1N 6N5, Canada

**Keywords:** adenovirus, fusion protein, cancer, therapy

## Abstract

Cancer is a devastating disease that affects millions of patients every year, and causes an enormous economic burden on the health care system and emotional burden on affected families. The first line of defense against solid tumors is usually extraction of the tumor, when possible, by surgical methods. In cases where solid tumors can not be safely removed, chemotherapy is often the first line of treatment. As metastatic cancers often become vigorously resistant to treatments, the development of novel, more potent and selective anti-cancer strategies is of great importance. Adenovirus (Ad) is the most commonly used virus in cancer clinical trials, however, regardless of the nature of the Ad-based therapeutic, complete responses to treatment remain rare. A number of pre-clinical studies have shown that, for all vector systems, viral spread throughout the tumor mass can be a major limiting factor for complete tumor elimination. By expressing exogenous cell-fusion proteins, many groups have shown improved spread of Ad-based vectors. This review summarizes the research done to examine the potency of Ad vectors expressing fusogenic proteins as anti-cancer therapeutics.

## 1. Introduction

Cancer is the second leading cause of death in high income countries, and the third leading cause of death in low and middle income countries, behind heart disease and infection [1]. Globally, in 2012, there were 14.1 million new cancer diagnoses, and 8.2 million cancer-related deaths. In the USA alone in 2011, the direct medical cost of cancer was a staggering 88.7 billion USD [2]. These numbers are expected to rise with global population and lifespan increase. Of all cancer cases discovered, 80% are classified as solid tumors [3]. When these tumors form in or near critical tissues, have diffuse borders, or occur in patients unable to tolerate surgery, they can be deemed inoperable. In such cases, chemotherapy (or other drug-based treatment) and radiation are the main modes of treatment. However, although these treatments can often reduce disease burden and increase lifespan, prognoses for inoperable cancers are often grim [4].

There are over 100 known major types of cancer that affect humans, each of which can have a diverse array of founding mutations, which influences pathology and treatment effectiveness [3]. A significant proportion of traditional chemotherapeutics aim to damage cellular DNA, and the differential response to DNA damage between normal and cancer cells is what underlies the effectiveness of many chemotherapeutic drugs. Upon DNA damage, normal cells undergo cell cycle arrest and engage the DNA damage repair (DDR) machinery prior to further cell replication and division. Cancerous cells often exhibit defects in the cell cycle checkpoints that would normally halt division or activate the DDR, and therefore these cancer cells will continue to replicate, most often dying in the process [5]. A major barrier to the success of these therapeutics is that the survival of a single cancerous cell can lead to re-emergence of the tumor and, similar to the evolution of antibiotic resistance, these cells often have or gain additional mutations that allow for resistance to future treatments [6]. This, and other mechanisms, can lead to the proliferation of cancer cells that are resistant to traditional chemotherapies, highlighting the need for new more potent and more specific cancer treatments for inoperable solid tumors [7].

One promising candidate therapy under development, viral gene therapy, has the capacity to offer cancer strain-specific treatments to counteract the exceptional diversity that exists within this disease group. Two basic strategies exist when using engineered viral vectors against cancer: use of the virus to deliver genes encoding therapeutic proteins to cancer cells, or engineering of the virus such that it selectively replicates in and kills cancer cells [8,9]. A wide array of viruses have been explored in preclinical and clinical studies, including vaccinia virus, herpes virus, vesicular stomatitis virus, and adenovirus [10,11,12,13]. Indeed, an oncolytic herpes simplex virus expressing granulocyte macrophage colony-stimulating factor (GMCSF) has been approved recently for clinical use in North America [14].

For one of the most commonly used gene therapy vectors, adenovirus (Ad), two Ad-based vectors have been approved for cancer treatment in China—a non-replicating Ad encoding p53, termed Gendicine, and an oncolytic Ad that selectively replicates in cancer cells, termed Oncorine [15,16]. In stage II/III clinical trials, intratumoral treatment with Gendicine with radiation treatment resulted in significant increases in both partial and complete responses, with a three-fold increase in complete responses versus radiation alone. Oncorine combination treatment with chemotherapy resulted in similar success rates with a 72.7% response rate versus 40.4% with chemotherapy alone. Ad vectors have been used extensively in molecular biology applications for many decades, to achieve high-level gene expression of a desired transgene in mammalian cells. As of July 2015, 22% (*n* = 506) of all human gene therapy clinical trials used Ad-based vectors to deliver a therapeutic gene of interest, with the vast majority of these trials directed towards treatment of cancer [17]. Adenovirus efficiently infects a wide variety of human cell types, regardless of cell cycle status, and has a relatively large cloning capacity [8]. These desirable qualities have led to a vast amount of research into their use as oncolytic vectors or as delivery vehicles for anti-cancer therapeutic genes. Unfortunately, many preclinical and clinical studies have shown that Ad-based therapeutics frequently mediate only a partial response [18,19], partly due to limitations of the vector system combined with complexities of the target tissue. A major barrier to anti-cancer efficacy for Ad, and many other therapeutic viruses, is efficient spread or distribution of virus throughout the tumor mass [20]. Tumors typically exhibit a relatively high internal pressure, which inhibits effective spread of virus when injected directly into the tumor mass [20]. Tumors also contain a large amount of connective tissue, further impeding virus spread [21]. Many approaches have been investigated to overcome this barrier, including the expression of junction opening peptides or enzymes capable of dissociating the extracellular matrix, or increasing the rate of viral lysis and spread by overexpression of native viral components such as the Ad death protein [22,23,24]. In addition to mechanical barriers to intratumoral spread, there are also several biochemical barriers. Tumors often express low levels of the main adenovirus receptor, the Coxsackievirus and Adenovirus Receptor (CAR), which can reduce the ability of Ad to bind to and enter the tumor cells [25,26]. Several methods of structural modification of Ad to increase CAR independent cellular uptake have been used, such as alterations to the fiber domain to increase efficiency of viral entry and fusion of single-domain antibodies to various capsid proteins [27,28,29]. Finally, pre-existing neutralizing Ad antibodies are detectable in 30% to 60% of the USA population, with higher rates in developing countries [30,31]. The existence of these neutralizing antibodies in combination with the efficiency of complement recognition of Ad can in theory cause rapid elimination of released virus following tumor cell lysis, further impeding spread throughout the tumor mass [32]. Interestingly, some studies suggest that presence or absence of pre-existing neutralizing Ad antibodies does not appear to affect treatment efficacy when Ad is delivered intratumorally [13,18].

An encouraging approach under investigation to enhance viral spread through a tumor involves heterologous expression of fusion-inducing proteins from the therapeutic vector. This approach has shown promise in not only increasing viral spread throughout the tumor mass, but also in facilitating activation of the host anti-tumor immune response. In this review, we will discuss recent studies in the development of vectors expressing fusogenic proteins for cancer therapy, focusing mainly on their use and delivery from Ad vectors.

## 2. Adenovirus Biology

**Figure 1:**
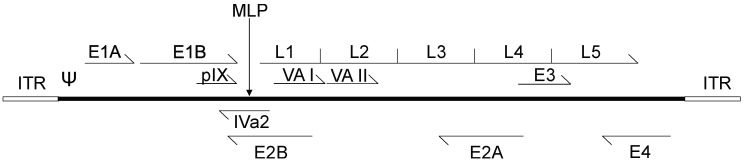
Schematic representation of the adenovirus genome. The inverted terminal repeat (ITR) sequences act as origins of viral replication, while Ψ represents the adenoviral packaging sequence. Adenovirus early transcripts (E1–E4) are transcribed upon viral entry, and expression of the intermediate transcripts pIX, IVa2, VA I and VA II follows. Activation of the major late promoter (MLP) occurs after the onset of adenovirus DNA replication, and generates a single transcript from which late transcription units, L1–L5, are spliced.

Although there are roughly 50 distinct serotypes of human *Adenoviridae*, Ad serotypes 2 and 5, both of subclass C, are the most well-studied [33]. The genomes of Ad2 and Ad5 are ~95% identical at the nucleotide level and, for the purpose of this review, will be considered experimentally identical. Adenoviruses are icosahedral, non-enveloped, double stranded DNA viruses of approximately 70–100 nm in diameter for the main “body” of the virus, with varying lengths of the viral attachment protein, fiber, protruding from the vertices of the icosahedron [33]. Adenoviruses display a naturally diverse tropism for tissue types, which varies between viral subtypes, however, in nature, Ad5 most often infects cells of the upper respiratory tract [34]. The Ad5 genome is roughly 36 kb and is transcribed in essentially two stages upon entry into the host cell (Figure 1) [35]. Soon after the Ad genome reaches the nucleus, the Ad early genes are expressed, including early region 1 (E1), E3 and E4, which function to activate other transcription units on the Ad genome as well as modulate the host cell cycle and immune responses [36]. The E2 region encodes proteins directly involved in Ad DNA replication, such as the virally-encoded DNA polymerase. Only after the onset of DNA replication is the major late promoter (MLP) activated, which results in the production of a large (roughly 26 kb) transcript which subsequently is processed through alternative splicing to produce the late transcripts late region 1 (L1) to L5 [37]. These late regions encode the structural components necessary for Ad assembly, including the Ad capsid proteins [38]. There are also four small products produced at intermediate/late times of infection, including the structural protein IX (pIX), and the IVa2 protein that helps package viral DNA into immature virions [39]. The other intermediate/late products, viral associated (VA) RNA I and II, inhibit activation of the interferon response, impede cellular micro-RNA processing, and may influence expression of host genes [40,41]. The viral DNA also contains the origins of replication, the inverted terminal repeats (ITR), which comprise ~100 bp at each end of the genome and the packaging sequence, which is ~150 bp in length and is located immediately adjacent to the left ITR [42].

The most extensively utilized class of Ad vector is the replication-defective E1-deleted Ad. The E1A and E1B proteins encoded within the E1 region are absolutely required for virus replication, and thus E1-deleted vectors are incapable of replicating in most cells [43], but can efficiently deliver their DNA to the nucleus of cells and express an encoded transgene placed under the control of a heterologous promoter. Early region 1-deleted Ad have been explored extensively as delivery vehicles for genes encoding therapeutic proteins with cancer-mitigating effects such as, for example, p53 or interleukin (IL)-12 [44,45].

For oncolytic, or conditionally replicating Ad, there are two major approaches to achieve cancer-specific replication. When viral genes encoding proteins essential for viral replication, (e.g., E1) are placed under the control of a tissue (cancer)-specific promoter (e.g., the prostate specific antigen promoter), some selectivity can be achieved in viral replication (e.g., only in prostate cancer cells) [46,47]. Alternatively, the virus can be attenuated or mutated such that it can only replicate in cancer cells with mutations in cellular pathways required to promote virus replication. For example, one of the functions of the Ad E1A protein is to interact with the retinoblastoma (Rb) family of pocket proteins, to promote cell cycle progression [48]. Deletion of a 24 amino acid segment of conserved region 2 of the E1A protein creates a molecule that can no longer interact with the pocket proteins, and virus with this mutation can no longer replicate efficiently in normal cells [49]. However, since the Rb pathway is mutated in many cancers, Ad vectors containing the E1AΔ24 mutation can selectively replicate in these cells but not normal tissue [50]. As stated above, two Ad-based cancer treatments are approved for use in China, one from each class of the above vector types—Gendicine and Oncorine (H101) [15,51]. Gendicine is a replication-deficient Ad expressing wild-type p53, which would reestablish expression of p53 in p53-deficient cancer cells, and Oncorine is a conditionally replicating Ad deleted of the E1B-55kD, which may also show specificity for cancers deficient in p53 [15,51].

As mentioned, a common problem with Ad is their restricted ability to disperse throughout a tumor, particularly following direct intratumoral injection [52,53,54]. Typically, Ad infection is limited to a 5 mm area surrounding the injection tract, which prevents efficient transgene product delivery to all cells of the tumor [19]. While this may not be a significant issue when Ad is used to deliver a gene for secreted proteins, such as cytokines, it may limit the efficacy of other types of therapeutic genes and proteins which require expression directly within the cell in order to exert their efficacy, such as, for example, the tumor suppressor p53. For oncolytic Ad vectors, preclinical studies have shown that complete tumor regression can be achieved with the oncolytic Ad ONYX-015 with initial infection of as little as 5% of the tumor cells, but these cells must be equally distributed throughout the tumor [52]. Consequently, distribution and efficacy can be improved through use of multiple, sequential injections of vector [52]. However, better, more effective approaches are required to enhance virus dispersion and spread within the tumor in order to achieve better efficacy.

## 3. Fusogenic Proteins

**Figure 2:**
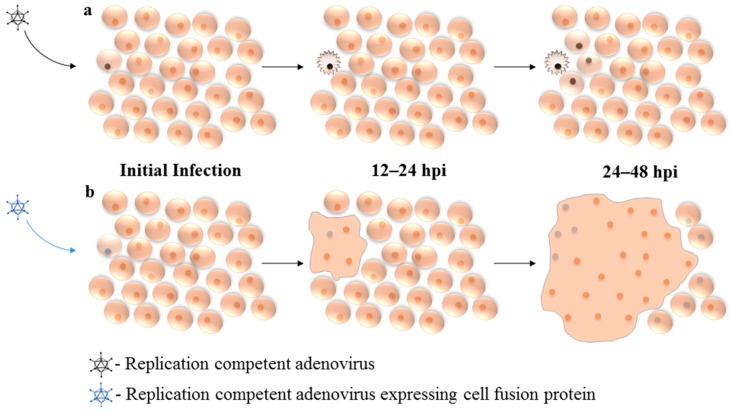
Mechanism of enhanced viral spread through expression of fusogenic protein: (**a**) Upon infection, replication-competent oncolytic adenovirus (Ad) must undergo productive replication and significantly damage the host cell in order to affect neighboring cells. (**b**) Cell-fusion proteins allow Ad to spread through densely packed cells with much greater efficiency, before replication-associated cell death or progeny release. Initial Infection: Virus enters host cell and initiates gene expression and early phase of replication. 12–24 hpi: Viral protein expression and progeny production. 24–48 hpi: Viral progeny released from initially infected cell, enter nearby cells. hpi: hours post-infection.

In an attempt to improve Ad vector distribution within the tumor, several research groups have explored whether inclusion of genes encoding fusogenic proteins can enhance therapeutic efficacy. Fusogenic proteins have the ability to promote cell–cell fusion, and thus their expression can promote lateral transfer of the virus or a co-expressed therapeutic gene product (Figure 2). The fusogenic proteins most often investigated tend to originate from enveloped viruses. These viruses, such as gibbon-ape leukemia virus (GALV), measles virus (MV) and respiratory syncytial virus (RSV), express membrane-bound fusogenic proteins within the viral envelope to facilitate viral entry into host cells [55,56,57]. There also exists a class of non-structural fusogenic proteins, the fusion associated small transmembrane (FAST) proteins, which are expressed from several species of non-enveloped *Orthoreovirus* [58]. Fusion associated small transmembrane proteins are not involved in virus entry but, instead, are believed to promote cell–cell fusion within the host as a means to increase viral lysis and progeny virus release.

Regardless of the virus of origin, the basic mechanism by which all fusogenic proteins function involves reducing the mechanical energy required for lipid bilayer fusion to occur [59]. However, the biochemical mechanism by which cell fusion occurs differs drastically between viral fusogenic proteins. Nevertheless, a few characteristics are shared by all fusogenic proteins. Syncytia, multinucleated cells caused by the fusion of multiple cellular membranes, are initially viable, and remain active both metabolically and transcriptionally [60]. In tissue culture studies in vitro, initially viable syncytia eventually lift from the dish and undergo a type of cell death that has not been definitively characterized [61]. Multiple conflicting studies have provided evidence in support of either apoptotic or non-apoptotic cell death [60,62,63]. Some studies point to a necrotic cell death mechanism, with expression of heat shock protein 70, heat shock protein 90kDa beta member 1 (gp96) and retention of inhibitor of kappa B (IκBα) in the cytoplasm, while others show elevation of cleaved caspase-3 and clear evidence of apoptotic DNA fragmentation [63,64]. However, the actual mechanism of cell death may be unique to fused cells, or even vary between fusogenic proteins.

During syncytium formation and associated cell death, blebbing at the cell surface results in the enhanced release of exosome-like particles referred to as syncytiosomes. These particles were shown to contain high levels of immune-stimulatory molecules, and lower levels of major histocompatibility complex I (MHC I) than exosomes naturally released from tumor cells [61]. Presence of these syncytiosomes promoted dendritic cell activation and resulted in activation of an anti-tumor T-cell response and tumor cell killing. Thus, fusogenic protein-induced formation of syncitia, and subsequent release of syncytiosomes, may enhance formation of anti-tumor immune responses, ultimately enhancing immune-mediated tumor rejection.

## 4. Expression of Fusogenic Proteins from Replication-Defective Adenovirus Vectors

The ability of viral fusogenic proteins to efficiently reduce cell viability while inciting a host anti-tumor response makes them excellent candidates as potential sole therapeutic molecules in non-replicating Ad vectors, or to enhance spread of Ad throughout the tumor mass in the context of oncolytic Ad vectors. A number of viral fusogenic proteins have been investigated for their anti-cancer effects in the context of both non-replicating and oncolytic Ad vector (Table 1), as discussed below.

### 4.1. Gibbon-Ape Leukemia Virus Fusogenic Membrane Glycoprotein

Gibbon ape leukemia virus (GaLV) is an enveloped C-type retrovirus, whose cellular target for attachment, PiT-1, is ubiquitously expressed in human tissues [83,84]. Through the examination of retroviral packaging cell lines, the GaLV fusion protein was shown to cause efficient human cell–cell fusion after truncation of the C-terminal R-peptide (a negative regulatory element) of the protein [85]. Bateman et al. showed that the GaLV fusogenic membrane glycoprotein (FMG) was able to significantly reduce viability of cancer cells in vitro and in vivo upon in situ transfection with a FMG expression plasmid [60]. Interestingly, mice vaccinated with tumor cells that had been pre-transfected with the FMG expression plasmid showed significant immune protection, compared to mice treated with cells which had been transfected with a plasmid encoding a non-fusogenic form of the protein, when subsequently challenged with subcutaneous tumors. As mice and hamsters do not express the PiT-1 receptor necessary for GaLV binding and fusion, these models could not adequately examine the safety profile of this therapeutic approach.

One concern with the use of fusion proteins is that fusion can occur in any cell type, including cancer and normal cells. To counteract this safety concern with the GaLV fusion protein, several groups attempted to restrict its fusogenic capacity specifically to cancer cells. One such proof-of-concept effort involved fusing Epidermal gowth factor (EGF) to the N-terminal targeting domain of the GaLV FMG via a factor Xa cleavage/linker domain, which was designated EXGaLV (EGF-X-GaLV) [86]. Incorporation of EXGaLV FMG into a retroviral vector (murine leukemia virus) limited infection to cells expressing the EGF receptor, and protease-mediated cleavage of the factor Xa domain resulted in rescue of viral infectivity [86]. Following this success, Johnson et al. sought to use a similar construct to target gliomas, which often overexpress the matrix metalloproteinase (MMP) enzymes MMP-2, MMP-9, MT-1 and MT-2, in stark contrast to the complete absence of MMPs in normal brain tissues [87]. The group used broad spectrum MMP-cleavable linkers to fuse the C-terminal domain of the cluster of differentiation 40 (CD40) ligand, to the N-terminus of the GaLV FMG. Addition of the fragment of the CD40 ligand prevents attachment through steric restriction, but would be cleaved from GaLV FMG in tumors expressing MMPs, thus permitting fusion. Transfection of a plasmid expressing this fusion protein into glioma cell lines in culture resulted in rampant cell fusion in cells with the highest expression of MMP, while no fusion was observed for normal human astrocyte (NHA) cells. When U87 glioblastoma cells were pre-transfected with a plasmid expressing the modified fusion protein and subsequently introduced into mice, MMP-mediated cleavage of the modified fusion protein was observed, along with fusion of the cancer cells and a concomitant significant reduction in tumor burden compared to animals receiving unmodified U87 cells. Unfortunately, the reduction of tumor volume was greatest in wild-type GaLV-transfected cells, suggesting an overall reduction in fusogenic capacity of the engineered protein. All of the above studies involved plasmid-mediated expression of the fusion protein; however, in general, plasmid delivery in vivo is relatively inefficient, resulting in reduced overall efficacy.

To improve the delivery efficiency of the targeted GaLV genes in vivo, multiple groups have utilized non-replicating Ad vectors. An Ad vector expressing the MMP-dependant GaLV construct (AdM40) was compared to a vector expressing a non-cleavable version of the protein (AdN40) in in vitro and in vivo models of glioma [65]. Studies in tissue culture showed that AdN40 was able to efficiently infect cells, but was unable to induce fusion. However, AdM40 induced significant fusion which was dependent on the expression of MMP, as co-administration of MMP inhibitors eliminated fusion. Upon injection of the viruses into subcutaneous U87 glioma xenograft tumors in BALB/c nude mice, a significant reduction in tumor volume was observed in AdM40-treated mice relative to untreated or AdN40-treated mice. Treatment with AdM40 also significantly improved survival, with 30% of treated mice showing complete eradication of tumors and survival at the end of the study period (50 days), compared to AdN40 which showed no improvement relative to untreated mice.

Another study used a non-replicating Ad expressing GaLV FMG under the control of a modified human heat-shock protein HSP70b promoter (HSE.70b), for thermal-mediated regulation of expression [66]. Hyperthermia can be easily induced in localized tissues, such as tumors, and has shown significant potential when used in conjunction with chemotherapy or radiotherapy [88]. The ability of this HSE.70b promoter to increase transcript levels 950-fold in response to heating enables this construct to have impressive potential to restrict transgene expression both temporally and spatially [66]. A stably transfected HT-1080 fibrosarcoma cell line expressing the transgene grew unimpeded until challenged with hyperthermia, which induced syncytium formation and a 70% reduction in cell viability. The median survival of mice bearing tumors composed of this cell line increased from 4.3 to 52 days when tumors were treated with mild hyperthermia (44 °C for 30 min), compared to an increase from 7.9 to 12.5 days for mice bearing tumors established using the parental HT-1080 cell line which were also treated with the same hyperthermia regime. Treatment of a variety of cell lines with a replication-deficient Ad containing the same expression cassette (Ad.HSE70b.GALV) showed no evidence of fusion until mild hyperthermia (43 °C for 30 min) was applied. Incorporation of the expression cassette into conditionally replicating Ad was suggested as the next step for follow-up on these encouraging results but, this has not yet been reported.

### 4.2. Measles Virus Fusion Proteins

Measles virus (MeV) is an enveloped, single-stranded RNA human pathogenic virus from the *Paramyxoviridae* family [89]. A hallmark of many *Paramyovirus* infections is the formation of syncytia in tissues harboring high-levels of virus [56]. Studies to develop antivirals have elucidated the structural components responsible for virus-host and host-host fusion observed in MeV infection. The MeV fusogenic membrane glycoprotein is a hetero-oligomeric protein composed of a tetramer of the H domain (attachment domain) complexed with a trimer of the F domain (fusion domain) [56]. Once associated in the endoplasmic reticulum, this complex of proteins is trafficked to the cell surface where it mediates both virus–host and host–host cell membrane fusion. The H domain is responsible for the MeV FMG cell specificity, and has two cellular protein targets: signaling lymphocyte activation molecule (SLAM, also known as CD150), or Nectin-4 (N4) [90]. Once the H domain binds to either of these targets, it mediates an irreversible conformational change in the F domain, which induces membrane curvature and ultimately membrane fusion [56].

In 1990, Alkhatib et al. published a study on their efforts to express the F domain of MeV from a non-replicating Ad [71]. The goal of the study was to explore the biochemical mechanism for fusion of the H/F FMG, however this was also the first proof-of-concept that heterologous expression of a viral FMG could generate functional fusogenic units and initiate cell–cell fusion in treated cells. Although the vector encoded only the F domain, expression of this protein induced syncytium formation in 293 and HeLa cell lines. Studies using plasmid-mediated expression of both the H and F domains showed that fusion could be achieved in a variety of glioma cell lines, and was most potent when the plasmids were administered at a 1:1 ratio [72]. To translate this work in vivo, two Ad vectors were constructed, AdF and AdH, which were subsequently tested in a U87 xenograft glioma tumor model in nude mice [72]. Virus was administered once daily for three days and, at the end of the study period (70 days), 100% of the AdH/AdF-treated mice were alive, compared to only 20% of the mice treated with Ad-green fluorescent protein (AdGFP). Interestingly, these researchers also tested the efficacy of a lentiviral vector expressing the GaLV FMG, and saw no significant reduction in tumor burden or increase in lifespan. However, of note, the dose of lentiviral vector was much lower than that of the Ad, due to the difficulty in generating high-titer lentivirus stocks. This observation highlights one of the advantages of Ad vectors—the ability to easily generate high-titer stocks of vector.

Due to the ubiquitous expression of SLAM and CD40 throughout the human body, several research groups have attempted to target the MeV FMG H domain to cells expressing particular cell surface markers. Single chain antibodies (scFvs) targeting carcinoembryonic antigen (CEA) or the myeloma marker CD38 were fused to the H domain of MeV FMG [91,92]. These mutant H domains conferred specificity upon ablation of the native H binding domains, and MeV expressing the mutant proteins were able to selectively fuse cells expressing the targeted binding proteins. In a follow-up study, the group fused a scFv recognizing EGF receptor (EGFR) to the H domain, and expressed this protein from a non-replicating Ad vector also expressing the F domain [90]. Upon infection in EGFR(−) Chinese Hamster Ovary (CHO) cells, this virus did not induce cell fusion. However, infection of genetically engineered EGFR(+) CHO cells, resulted in widespread fusion. Taken together, these studies clearly show that some fusogenic proteins can be altered to decrease “generalized” fusion, and achieve cell selective fusion using hybrid proteins.

To enhance immune responses to the treated tumor cells, and essentially promote in situ vaccination, an Ad vector expressing the MeV H/F FMGs (Ad.MV-H/F) was tested alone and in combination with Ad vectors expressing various cytokines IL-2, IL-12, IL-18, IL-21 or granulocyte-macrophage colony-stimulating factor (GM-CSF) [73]. Previous studies had shown that expression of an FMG could improve the immunogenicity of a weakly allogenic melanoma vaccine [93], possibly due to the enhanced release of syncytiosomes from the fused cells, and subsequent cross-presentation of tumor-associated antigens to dendritic cells [61]. Mice bearing MC38 syngeneic colorectal tumors in both left and right hind flanks received intratumoral injection of Ad.MV-H/F in combination with the various cytokines in only one of the two tumors [73]. Combination treatment led to an 87%–98% reduction in injected tumor size at the end of the study, compared to treatment with Ad.MV-H/F (51% reduction) or Ad-cytokines alone (1%–40% reduction). Impressively, contralateral, uninjected tumors showed similar response rates, with a significantly greater response in mice treated with Ad.MV-H/F in combination with cytokines (85% reduction in tumor size) compared with treated with either Ad.MV/H-F (54% reduction) or Ad expressing cytokines alone (8% to 46%). Both the injected and non-injected tumors showed high levels of macrophage infiltration when Ad.MV-H/F was combined with the cytokine-expressing vectors.

### 4.3. Other Fusogenic Envelope Proteins

In 2007, Hoffman et al. examined the therapeutic efficacy of Ad vectors expressing the RSV fusion peptide RSV-F or vesicular stomatitis virus (VSV) fusion peptide VSV-G [77,78]. Reduction in tumor growth in mice treated with Ad.RSV-F was similar to that observed for Ad.MV-H/F noted above. Although Ad.VSV-G also mediated a significant anti-tumor effect, it was less effective than Ad vector expressing either of the other two fusogenic proteins. Another potential FMG for immune stimulation is the simian virus SV5-F protein. The SV5-F protein is a potent antigen, and displayed promise in early cell-culture models of cancer [79]; however, no follow up experiments were performed to further explore its anticancer capacity in vivo. Considering these results, the measles virus fusion proteins, as well as other similar fusogenic glycoproteins, offer considerable promise in arming oncolytic Ad, but this remains to be tested experimentally.

### 4.4. Fusion Associated Small Transmembrane Proteins

Unlike the FMGs discussed above, the FAST proteins of the *Reoviridae* family were discovered in non-enveloped viruses [58]. The primary function of all of the FMGs described above is to mediate fusion of the viral membrane to the host membrane during virus internalization. Non-enveloped viruses do not enter the cell through fusion, therefore the primary function of the non-structural FAST proteins is believed to be to aid in lateral transmission of the virus.

As their name implies, FAST proteins are small in size, and include the 10 kDa proteins from Nelson Bay and avian reoviruses, the 14 kDa reptilian reovirus protein and the 15 kDa baboon reovirus protein [58]. Their small size makes them excellent candidates for use in oncolytic viruses, since they readily fall within the cloning capacity of most vector systems. In the context of Ad, use of such small fusogenic proteins means that there would be sufficient remaining cloning capacity to accommodate a tissue-specific promoter to confer tumor selectivity or a second transgene (e.g., a cytokine to enhance immune stimulation) in the same vector. Early studies showed that transfection of a plasmid expressing the p14 FAST protein induced extensive syncytium formation and apoptosis, once again suggesting that these proteins may be ideal as anti-cancer therapeutics [63].

To evaluate the ability of the p14 FAST protein to elicit an anti-cancer effect, Brown et al. published work examining expression of the p14 FAST protein from an otherwise wild-type VSV [94]. In cell culture, this VSV/FAST recombinant virus induced the formation of large syncytia with no deleterious effect on VSV replication. After intravenous injection, the VSV/FAST virus entered the central nervous system of BALB/c mice more rapidly and replicated to a higher titer than VSV/GFP (1 to 4 logs higher between 24 and 96 h post infection). When delivered intranasally, VSV/FAST also caused clinical pathogenesis (hind-limb paralysis) at a much lower dose than VSV/GFP (1 × 10^7^ plaque-forming unit (PFU) vs. 5 × 10^8^ PFU per mouse, respectively). These results indicate that the p14 FAST protein can act as a virulence factor in non-endogenous viruses. The efficacy of FAST was also examined in a VSV derivative that is selective for replication in cancer cells, VSVΔ51 [95,96]. VSVΔ51/FAST, was tested for oncolytic potency in combination with VVDD, a double-deleted vaccinia virus whose replication is restricted to cells that overexpress the E2F transcription factor [96]. The two viruses were chosen for their complementary effects; VVDD naturally inhibits production of type I interferons (IFNs), while VSVΔ51 is sensitive to this antiviral pathway [95,97]. Compared to treatment with VVDD/VSVΔ51, VVDD/VSVΔ51FAST caused increased cell killing (shown by crystal violet assay) and a 100-fold increase in VVDD production. These results suggest that the p14 FAST protein can be used in non-endogenous oncolytic viruses to enhance efficiency.

Due to the attractive properties of p14 FAST, our lab created a non-replicating Ad, AdFAST, expressing the p14 FAST protein from the high-activity cytomegalovirus immediate early enhancer/promoter (CMV) replacing the E1 region [62]. In A549 lung adenocarcinoma cells, AdFAST efficiently caused formation of syncytia at high multiplicity of infection (MOI). Syncytium formation was shown to reduce metabolic activity compared to a control Ad vector lacking a transgene. AdFAST-treated cells showed a significant increase in cleaved caspase-3 compared to cells treated with control virus, suggesting the AdFAST induced apoptosis in the treated cells. Unfortunately, these promising in vitro results did not translate in vivo, as immunodeficient mice bearing subcutaneous A549 tumors did not show improved tumor regression or prolonged lifespan when treated with AdFAST compared to animals treated with control virus or vehicle. AdFAST was also tested in an immunocompetent, syngeneic mouse model of cancer but, unfortunately, no increase in treated mouse survival or reduction in tumor growth was observed [80]. Studies in vitro in 293 cells, in which the AdFAST virus can replicate due to complementation of the E1-deletion in this cell line, showed that extensive fusion and a decrease in metabolic activity could be achieved at a very low MOI [62]. These observations suggest that the ineffectiveness of AdFAST in the A549 model may be due to inefficient levels of FAST protein expression, and that FAST may be more effective when included in an oncolytic Ad vector. Replication of the oncolytic Ad would dramatically increase the template copy number of the FAST gene within the tumor cell, resulting in enhanced FAST protein expression and, likely, improved tumor regression.

## 5. *Trans*-Complementing Adenovirus Systems for Expression of Fusion Proteins

Despite the relatively large cloning capacity of Ad, it can be difficult to encode traditional FMGs in replication competent Ad due to DNA packaging limits [98,99]. The Ad capsid can accommodate about 105% of the normal genome length, giving a cloning capacity of about 1.8 kb in a vector that does not contain any compensatory deletions. Most traditional FMGs are rather large proteins and often require multiple subunits [55,71], and either can not be accommodated in a single Ad vector (especially if the vector retains the E1 region, such as in most oncolytic Ad) or preclude the ability to incorporate additional therapeutic genes (such as immune modulators). Chen et al. (2011) circumvented this issue by using tumors based on 293 cells, which complement the E1-deletion in traditional Ad vectors permitting replication in these E1+ tumors [67]. The authors explored the efficacy of an E1-deleted Ad vector encoding the GaLV FMG under regulation by the human endothelial receptor tyrosine kinase (eTie1) promoter, which is a vascular endothelial specific promoter activated in proliferating endothelium, such as that found during tumor angiogenesis [67]. The study sought to improve Ad escape from the vasculature of the tumor following intravenous (i.v.) injection. Quantitative polymerase chain reaction (qPCR) analysis of xenograft tissue extracted from tumor-bearing mice treated with Ad-eTie1-GALV showed three-fold higher genome copy numbers compared to animals that had received a control Ad lacking the fusion gene. Furthermore, hybrid cells derived from the fusion of the 293 cells and endothelial cells were observed in the tumor periphery, suggesting improved escape of Ad from the vasculature mediated by virus-induced fusion.

Vile et al. overcame the cloning limitations in Ad vectors by using plasmid-mediated expression of the FMG in combination with delivery of a therapeutic, oncolytic Ad [68]. Plasmid-mediated expression of GaLV FMG in combination with delivery of Ad5 wild-type was able to eradicate slow-growing tumors in 4 of 10 treated mice, leading to long term survival [68]. In faster-growing tumors, expression of the FMG and viral replication could not keep tumor growth in check and survival increases were minimal. This study also showed that co-expression of the FMG with replicating Ad increased viral release and spread through a monolayer of cells in vitro. In this co-culture experiment, human glioma U87 tumor cells were infected with AdGFP and Ad5 wild-type (WT), and subsequently transfected with a plasmid expressing GaLV FMG. When these cells were co-cultured with mouse B16 cells, which lack the PiT-1 receptor for GaLV (and therefore can not fuse to U87 cells), AdGFP spread to the B16 cells at a higher frequency relative to un-transfected cells, suggesting a fusion-independent increase in viral spread induced by enhanced release of the virus in the FMG-expressing cells.

Another method to circumvent the cloning restrictions of Ad is through *trans*-complementation. When a host cell is infected with multiple Ad strains, the proteins from both viruses are expressed, and complementation of missing genes can occur. Thus, when cells are co-infected with an E1-deleted Ad carrying a copy of an FMG gene and a replication competent Ad carrying the E1 sequences necessary for replication, viral progeny can be produced from both viruses, and viable Ad carrying the FMG transgene can spread to new cells [100]. An E1-deleted Ad expressing the MeV fusion proteins (Ad.H/F) was tested in combination with the replication-restricted vector Ad.COX∙MK [74,75]. This latter vector put two regions critical for Ad replication, the E1 and the E4 regions, under the control of the cyclooxygenase-2 and midkine promoters, respectively, restricting replication of the virus to pancreatic and colon cancer cells. This combination of vectors was tested both in xenograft models of pancreatic and colorectal cancer, and in combination with multiple chemotherapeutics. In human pancreatic subcutaneous xenografts (CFPAC-1), the triple-treatment combination of Ad.H/F, Ad.COX∙MK and gemcitabine, an antineoplastic chemotherapeutic, resulted in the greatest reduction in tumor volume compared to all other combinations. In intraperitoneal xenografts, which mimic metastatic disease, the combination of Ad.H/F and Ad.COX∙MK resulted in one long-term survivor (alive at the end of the observation period, 120 days) of eight treated mice, while the triple combination resulted in three long-term survivors. No other treatment group had survivors. When tested in a colorectal cancer xenograft model in mice, a similar trend was observed when the viruses were combined with the chemotherapeutic FOLFOX (folinic acid, 5-fluorouracil and oxaliplatin). The group also found a synergistic effect between an Ad5 mutant bearing Ad35 fiber (for improved tumor penetrance), and Ad.H/F in melanoma models of cancer [76]. Taken together, these results show that expression of fusogenic proteins can be used to increase the potency of replication-competent Ad.

## 6. Expression of Fusogenic Proteins from Replication Competent Adenovirus

To date, few groups have successfully shown expression of a fusogenic protein from a replication competent Ad, mainly due to two barriers to vector propagation. First, as discussed above, DNA packaging limitations of the Ad capsid limits the amount of “extra” DNA that can be included in the vector, and genes for fusogenic proteins tend to be large. Second, high-level expression of the fusogenic protein during vector propagation may lead to premature cell death, resulting in an inability to effectively grow stocks of the virus. In 1989, Dewar et al. used an E1-competent, E3-deleted Ad to express the human immunodeficiency virus (HIV) envelope glycoproteins (Ad5env) [81], which was the first evidence that Ad replication was compatible with cell–cell fusion. Twelve years later, Li et al. showed that expression of the HIV envelope glycoprotein from this same virus increased viral release, spread, and replication, compared to wildtype Ad5, in an HeLa-CD4+ cell culture model, which is permissive for HIV envelope protein-mediated fusion [82].

As mentioned, incorporating GaLV and other FMGs into conditionally replicating Ad has proven difficult, as FMGs often cause cell death prior to generation of high titers of virus. Some evidence suggests that tetracycline regulation of toxic transgenes could help to bypass this problem by restricting expression of the transgene during viral propagation in vitro [101]. Another strategy to circumvent this issue is to place the FMG under regulation by the Ad MLP, thus allowing the virus to begin replication prior to expression of the lethal fusogenic protein [69]. This strategy was used to generate a conditionally replicating virus containing the GaLV FMG under regulation of the MLP, with E1 expression controlled by a tumor-selective promoter containing eight E2F-binding sites and one specificity protein 1 (Sp1)-binding, designated ICOVIR16. This virus showed potent anti-tumor activity in mouse xenograft subcutaneous models of pancreatic cancer (NP18) and melanoma (SkMe128) [70]. ICOVIR16 was significantly better than its parent virus, ICOVIR15, which contains only the regulated E1 expression and lacks an FMG, at reducing tumor volume following intratumoral injection. In addition, the virus also showed anti-tumor efficacy following systemic administration, which would be important for potentially reaching undetected metastases. As mouse cells are non-permissive for Ad replication, a Syrian hamster model was used to assess toxicity of the armed virus. Importantly, arming of ICOVIR16 did not increase the systemic toxicity of the virus, suggesting that the presence of the fusogenic protein did not affect tumor-specific replication, thus offering a promising platform for future studies. Therefore, equipping selectively replicating Ad with fusogenic proteins is a powerful strategy to increase antitumor activity with no apparent increase in toxicity.

## 7. Conclusions

The promising results from early experiments with armed Ad in the fight against inoperable tumors has led to the development of a variety of strategies to combat this devastating disease. Expression of fusogenic proteins from viral vectors can not only act as an effective sole therapeutic, directly resulting in cancer cell killing, but can also enhance virus spread between cancer cells in a tumor mass, and promote release of syncytiosomes that may augment the anti-tumor immune response. It is likely that these fusogenic proteins are best suited for use in the context of conditionally replicating Ad vectors, to take full advantage of the ability of fusogenic proteins to aid in virus spread and dispersion in a tumor. Continued research into new and more potent fusogenic proteins, as well as development of more specific and active conditionally replicating Ad vectors will undoubtedly further enhance this very promising area of anti-cancer therapeutic research.

## Figures and Tables

**Table 1:** Adenovirus expressing fusogenic proteins.

Origin of Fusion Protein	Replication-Defective Ad Vector	Replication-Competent Ad Vector	Reference
Gibbon-Ape Leukemia Virus	✓	-	[65,66]
	-	✓	[67] ^1^, [68] ^2^, [69,70]
Measles Virus	✓	-	[71,72,73]
	-	✓	[74,75,76] ^3^
Respiratory Syncytial Virus	✓	-	[77]
Vesicular Stomatitis Virus	✓	-	[78]
Simian Virus 5	✓	-	[79]
Reptilian Reovirus	✓	-	[62,80]
Human Immunodeficiency Virus	-	✓	[81,82]

^1^ Early region 1 (E1)-deleted viruses used in E1-expressing 293 tumors; ^2^ Plasmid expressing fusion protein combined with E1-competent Ad; ^3^ E1-deleted Ad trans-complemented with E1-competent Ad.
